# Effects of visual disruption on static and dynamic postural control in people with and without chronic ankle instability

**DOI:** 10.3389/fbioe.2024.1499684

**Published:** 2024-11-05

**Authors:** Yushan Miao, Yubin Ge, Dongmei Wang, Dewei Mao, Qipeng Song, Rentana Wu

**Affiliations:** ^1^ Graduate school, Shandong Sport University, Jinan, China; ^2^ School of Human Movement Science, Beijing Sport University, Beijing, China; ^3^ School of Sports and Health, Shandong Sport University, Jinan, China; ^4^ Division of Physical Education, The Chinese University of Hong Kong, Shenzhen, China; ^5^ School of Arts, Shanghai University of Sport, Shanghai, China

**Keywords:** ankle sprain, sensory feedback, balance control, vision, rehabilitation

## Abstract

**Introduction:**

Chronic Ankle Instability (CAI) is a chronic syndrome resulting from repeated ankle sprains that lead to persistent dysfunction.the purpose of this study is to determine whether visual disruption could influence static and dynamic postural control in people with and without chronic ankle instability (CAI), with the objective of gaining a comprehensive understanding of the interactions between visual inputs and postural control.

**Methods:**

Thirty people with CAI (21 males and 9 females, age = 22.0 ± 1.8 years, height = 174.4 ± 10.2 cm, body mass = 72.5 ± 15.4 kg; Cumberland Ankle Instability Tool (CAIT) score = 19.7 ± 1.8) and twenty-nine without CAI (24 males and 5 females, age = 22.9 ± 1.6 years, height = 172.8 ± 8.0 cm, body mass = 69.0 ± 11.3 kg; CAIT score = 29.0 ± 0.7) were recruited. Their static and dynamic postural control was measured in two conditions with or without visual disruption, simulated using stroboscopic glasses. Static postural control was measured during single-limb standing and represented by root mean square (RMS) of the plantar center of pressure (CoP), dynamic postural control was measured during a Y-balance test and represented by the relative reach distance. Two-way mixed ANOVA (between group: CAI vs non-CAI, within group: normal vision vs visual disruption) was used to analyze data.

**Results and discussion:**

Significant interactions were detected in the CoP-RMS in the anteroposterior (AP) (*p* = 0.021, η^2^_p_ = 0.090) and mediolateral (ML) (*p* < 0.001, η^2^_p_ = 0.208) directions, and the relative reach distances in the posteromedial (PM) *p* = 0.023, η^2^_p_ = 0.088) and posterolateral (PL) (*p* = 0.009, η^2^_p_ = 0.113) directions, from normal vision to visual disruption. The CoP-RMS in the AP and ML directions significantly increased and the relative reach distances in the PM and PL directions significantly decreased in people with CAI while remaining unchanged in those without CAI. People with CAI are susceptible to visual disruption on postural control, highlighting the importance of visual input in maintaining stable posture in this population.

## 1 Introduction

Acute ankle sprains are among the most prevalent musculoskeletal injuries (Waterman et al., 2010). Up to 70% of people who experience an acute lateral ankle sprain may develop chronic ankle instability (CAI) within a year following the initial injury (Herzog et al., 2019). Annually, the United States spends approximately 152 million USD on the treatment of ankle sprains (Feger et al., 2017).

The acute ankle sprains experienced by people with CAI may impact their static and dynamic postural control (Sierra-Guzmán et al., 2018). Diminished postural control is associated with falls (Moncada and Mire, 2017) and injuries (Hrysomallis, 2007; Paterno et al., 2010), particularly among those with CAI (Prawiradilaga et al., 2020). Static balance control, typically quantified by the root mean square (RMS) of plantar center of pressure (CoP) during single-leg standing (Song et al., 2021), may be compromised by an acute ankle sprain, due to the decreased tactile sensation that follows the injury (Liu et al., 2024). Similarly, dynamic postural control, often assessed by the relative reach distance during the Y-balance test (YBT) (Sierra-Guzmán et al., 2018), may also be adversely affected by an acute ankle sprain. This may be attributed to the reduction in lower extremity muscle strength after the injury (Hou et al., 2020).

The static and dynamic postural control of people with CAI may be more significantly influenced by visual disruption than those without CAI. The visual system plays a pivotal role in enhancing postural control, as it is adept at object motion perception and recognition, and is highly sensitive to dynamic scenes (Guerraz and Bronstein, 2008). A study has demonstrated reduced ankle proprioception among people with CAI (Xue et al., 2021), potentially necessitating a greater reliance on visual cues to maintain postural control (Song et al., 2016). People inevitably encounter environments or conditions that cause visual disruption, such as reduced visual stimulation due to dim lighting (Honeine and Schieppati, 2014) or decreased visual perception as a result of aging (Nguyen et al., 2021), people with CAI may particularly vulnerable due to their existing proprioception deficits, which may be used to compensate for other decreased sensations (Liu et al., 2023), like vision.

The specific impact of visual disruption on their postural control has not been fully understood. Further exploration in this area is crucial for gaining a comprehensive understanding of the complex interaction between visual input and postural control in this specific population. This study aims to determine whether visual disruption would affect static and dynamic postural control in people with and without CAI. We hypothesized that compared to those without CAI, visual disruption might result in #1. greater increases in the CoP-RMS; and #2. greater decreases in the relative reach distance during the Y balance test in people with CAI.

## 2 Methods

### 2.1 Participants

To the best of our knowledge, no prior studies have examined the impact of visual disruption on the CoP-RMS during single-limb standing, and the relative reach distance during the Y Balance Test in people with and without CAI. Data from our pilot study have been used for sample size estimating, where 3 people with CAI and another 3 without CAI participated. The effect size (η^2^_p_) for the group-by-condition interaction pertaining to the CoP-RMS and relative reach distance of YBT were 0.064 and 0.037. An *a priori* power analysis utilizing G*Power (3.1, Universität Düsseldorf, Düsseldorf, Germany) revealed that a sample size of 54 participants (with 27 participants in each group) would be necessary to achieve an alpha level of 0.05 and a statistical power of 0.80.

Thirty people with CAI (9 females and 21 males, aged 22.0 ± 1.8 years, with a height of 174.4 ± 10.2 cm and a body mass of 72.5 ± 15.4 kg; Cumberland Ankle Instability Tool (CAIT) score = 19.7 ± 1.8) and twenty-nine people without CAI (5 females and 24 males, aged 22.9 ± 1.6 years, with a height of 172.8 ± 8.0 cm and a body mass of 69.0 ± 11.3 kg; CAIT score = 29.0 ± 0.7) participated in this study. The inclusion criteria for people with CAI adhered to the guidelines by the International Ankle Consortium (Gribble et al., 2013), which included: (a) experiencing at least one lateral ankle sprain 12 months prior to enrollment, leading to cessation of physical activity for at least 1 day; (b) experiencing at least two lateral ankle sprains; (c) experiencing at least two episodes of ankle “giving way” within the 6 months preceding enrollment; and (d) a CAIT score of less than 24. The inclusion criteria for people without CAI were: (a) matching the CAI participants in terms of gender, age (±3 years), height (±5 cm), and weight (±5 kg); (b) having no history of lateral ankle sprains; and (c) a CAIT score of 28 or higher. The exclusion criteria for both groups were (Song et al., 2017): (a) having a lower extremity fracture or surgery; (b) sustaining an acute injury to the lower extremity within the last 3 months; (c) having neurological disorders, diabetes mellitus, or vestibular disorders; and (d) having bilateral CAI. This study received approval from the ethics committee of Shandong Sport University (No. 2022044), and all participants provided written informed consent.

### 2.2 Protocols

The participants wore form-fitting T-shirts and shorts, with their tested limbs designated as the affected limbs for people with CAI and the corresponding limbs on the same side for those without CAI. Each participant underwent two tests: the single-limb standing test and the YBT, under both normal vision and visual disruption conditions. We randomly tested the single-limb standing test and Y-balance test with normal vision and visual disruption by using a computer-generated randomized sequence. Visual disruption was simulated using stroboscopic glasses (Sibokeji, Zhuhai, China) at 3 Hz, alternating between 0.100 s of clarity and 0.233 s of opacity (Han et al., 2022).

#### 2.2.1 Single-limb standing test

The static balance control was quantified using the single-limb standing test. In this test, participants stood with their tested foot placed on a force platform (AMTI, BP600900, United States), maintaining their body as still as possible with their hands resting on their hips. The untested hip was flexed at 30° and the knee at 45°, and this position was held for 30 s under normal vision and visual disruption conditions. Throughout the test, participants fixated their gaze on a black dot positioned 1.8 m above a wall located 2.5 m away (Sun et al., 2018). The CoP data were recorded for approximately 30 s at a 1,000 Hz. One trial was conducted separately under normal vision and visual disruption conditions, separately.

#### 2.2.2 Y-balance test

The Y-Balance Test Kit was utilized to evaluate the participants’ postural control capabilities (Plisky et al., 2009). This comprehensive kit consists of a central plate and three extendable tubes positioned in the anterior (ANT), posteromedial (PM), and posterolateral (PL) directions, each tube terminating in a unique plastic plate. During the assessment, participants stood barefoot on the central plastic plate, with their tested leg as the base of support, and pushed each of the three movable plates as far as possible with their non-tested leg. Each direction (ANT, PM, PL) was tested separately, with three trials conducted in each under normal vision and visual disruption conditions (Shaffer et al., 2013), the mean value of the three trials for each direction was then calculated for data analysis.

### 2.3 Data reduction

During the single-limb standing test, the CoP data were subjected to low-pass filtering at a cutoff frequency of 10 Hz (Zéronian et al., 2021) for a duration of 10 s, commencing approximately 3–5 s after the onset of the standing task (Zéronian et al., 2021). Equation 1 was used to calculate these filtered data to calculate the CoP-RMS.

$$\text{RMS}=\sqrt{\frac{1}{n}\sum_{i=1}^{n}{\left(x_{i}-\bar{x}\right)}^{2}}$$ (1)

where $x_{i}$ donates the CoP position for each measurement; $\bar{x}$ donates the average of all measurements; n donates the number of measurements

During the Y-balance test, participants’ leg lengths were measured from the anterior-superior iliac spine to the distal end of the medial malleolus. The relative reach distance was normalized by leg length using Equation 2 (Johnston et al., 2018):

Relative reach distance=reach distance/leg length×100% (2)

### 2.4 Statistics

Data were analyzed using SPSS (26.0, IBM, New York, United States) and G*Power (3.1, Universität Düsseldorf, Düsseldorf, German). Shapiro-Wilk tests were used to verify the normality of the data. Two-way mixed-design ANOVAs, distinguishing between-group factors (CAI vs. non-CAI) and within-group factors (normal vision vs. visual disruption), were utilized to ascertain the main effects of group and condition, as well as the group-by-condition interactions. If significant interactions were identified, simple effects analyses were employed to perform pairwise comparisons. When the data did not follow a normal distribution, the non-parametric Mann-Whitney U test was used to compare the differences between the two groups under normal vision and visual disruption conditions. Partial eta squared (η^2^_p_) was used to indicate the effect size of the two-way ANOVA’s interactions and main effects with the thresholds: 0.01–0.06 for small, 0.06–0.14 for moderate, and >0.14 for large effect size (Richardson, 2011). Cohen’s *d* was used to indicate the effect size of *post hoc* pairwise comparison with the thresholds: <0.20 for trivial, 0.21–0.50 for small, 0.51–0.80 for medium, and >0.81 for large effect size (Cohen, 1988). Data are presented as mean ± standard deviation and the significance level was set at 0.05.

## 3 Results

As shown in Table 1, a group-by-condition interaction was detected in CoP-RMS in the anteroposterior (AP) (*p* = 0.021, η^2^_p_ = 0.090) and mediolateral (ML) (*p* < 0.001, η^2^_p_ = 0.208) directions. From normal vision to visual disruption conditions, the CoP-RMS in the AP (normal vision = 9.36 ± 2.19 mm, visual disruption = 12.64 ± 7.05 mm, *p* = 0.005, *d* = 0.63) and ML (normal vision = 7.47 ± 2.01 mm, visual disruption = 8.54 ± 2.28 mm, *p* < 0.001, *d* = 0.50) directions significantly increased in people with CAI while remained unchanged in those without CAI.

**TABLE 1:** Root mean square of center of pressure in the single-limb standing test.

	CAI (N = 30)		Non-CAI (N = 29)		Group effect		Condition effect		Interaction	
	Normal vision	Visual disruption	Normal vision	Visual disruption	p	η^2^_p_	p	η^2^_p_	p	η^2^_p_
RMS_AP_ (mm)	9.36 ± 2.19	12.64 ± 7.05^a^	9.39 ± 2.42	8.91 ± 6.15	0.073	0.050	0.083	0.052	0.021	0.090
RMS_ML_ (mm)	7.47 ± 2.01	8.54 ± 2.28^a^	7.79 ± 1.66	7.41 ± 1.43	0.378	0.014	0.071	0.056	<0.001	0.208

RMS_AP/ML_: the root mean square of center of pressure in the anteroposterior or mediolateral directions.

^a^ denotes a significant difference compared with Visual disruption conditions in people with CAI.

As shown in Table 2, significant group-by-condition interactions were detected in the relative reach distances in PM (*p* = 0.023, η^2^_p_ = 0.088) and PL (*p* = 0.009, η^2^_p_ = 0.113) directions. From normal vision to visual disruption conditions, the relative reach distance in the PM (normal vision = 107.0 ± 11.6%, visual disruption = 110.1 ± 12.2%, *p* = 0.046, *d* = 0.26) and PL directions (normal vision = 111.1 ± 10.8%, visual disruption = 114.4 ± 9.0%, *p* = 0.026, *d* = 0.33) significantly decreased in people with CAI while remained unchanged in those without CAI. In addition, significant group main effects were detected. Compared with people without CAI, those with CAI have a significantly shorter relative reach distance of YBT in the PM (*p* = 0.003, η^2^_p_ = 0.141) and PL (*p* = <0.001, η^2^_p_ = 0.194) directions.

**TABLE 2:** Relative reach distance in the Y-balance test.

	CAI (N = 30)		Non-CAI (N = 29)		Group effect		Condition effect		Interaction	
	Normal vision	Visual disruption	Normal vision	Visual disruption	p	η^2^_p_	p	η^2^_p_	p	η^2^_p_
Anterior (LL%)	68.3 ± 9.0	66.2 ± 9.1	74.1 ± 8.5	72.7 ± 7.7	0.006	0.123	0.003	0.147	0.480	0.009
Posteromedial (LL%)	110.1 ± 12.2	107.0 ± 11.6^a^	117.9 ± 13.4	119.9 ± 16.6	0.003	0.141	0.607	0.005	0.023	0.088
Posterolateral (LL%)	114.4 ± 9.0	111.1 ± 10.8^a^	122.9 ± 14.0	125.2 ± 15.1	<0.001	0.194	0.613	0.005	0.009	0.113

Anterior, posteromedial, and posterolateral represent the relative reach distances in the anterior, posteromedial, and posterolateral directions.

LL, represents leg length.

^a^ denotes a significant difference compared with visual disruption conditions in people with CAI.

## 4 Discussion

This study aimed to investigate the impact of visual disruption on static and dynamic postural control in people with and without CAI. Our results supported hypotheses #1 and #2. Specifically, visual disruption significantly increased CoP-RMS, and significantly decreased the relative reach distance in the YBT among people with CAI, whereas these effects were not observed in people without CAI.

Our findings reveal that visual disruption leads to a decrease in static postural control, as indicated by CoP-RMS, in people with CAI, whereas this effect is not observed in those without CAI. This reduction in static postural control is often interpreted as a sign of decreased automation of static posture control (Roerdink et al., 2011; Rizzato et al., 2023). Our finding is consistent with previous studies. Song et al. investigated the effect of closed eyes on the CoP displacement during single-limb standing test in people with CAI, and their results showed that with the reduction of visual information, the static postural control during single-limb standing test in people with CAI decreased (Song et al., 2017). Furthermore, Lee et al. showed a significant decrease in static postural control in healthy people when both somatosensory and visual disruption were present, whereas there was no significant change when only visual disruption was present (Lee et al., 2022).

We propose that the impact of visual disruption on people with CAI stems from their limited ability to allocate additional attentional resources towards maintaining stability. Attentional resources are crucial for maintaining physical stability and integrating sensory information (McDowd, 2007; Katsuki and Constantinidis, 2014). When sensory information is inadequate or inaccurate, people require more attentional resources to process this information compared with people without sensory deficits (Shumway-Cook and Woollacott, 2000; Redfern et al., 2001a; Teasdale and Simoneau, 2001). Given that people with CAI require more attentional resources to process their compromised sensory information (Song et al., 2017; Terada et al., 2019), visual disruption further increases the demand for attentional resources to maintain stability, ultimately leading to a deficit in the resources allocated to physical stability and a subsequent decrease in static postural control. Several pieces of evidence support our hypothesis. Firstly, reduced relevant sensory information causes decreased static postural control in people with CAI (Song et al., 2017). Secondly, when visual information is reduced, people with impaired or absent sensory functions tend to be more concerned about maintaining physical stability (Brown et al., 1999; Lindenberger et al., 2000; Rankin et al., 2000; Marchese et al., 2003; Redfern et al., 2004). These findings suggest that the visual disruption in our study may have disproportionately affected people with CAI, leading to a reduction in static postural control and potentially increasing their risk of injury.

Our results further demonstrated that visual disruption significantly impaired dynamic postural control in people with CAI, whereas it had no discernible effect on those without CAI, suggesting a heightened reliance on vision for maintaining stability among those with CAI. A previous study has shown that people with CAI are more visually dependent than those without CAI in performing the dynamic task of one-legged jumping and that somatosensory sensations other than vision are not sufficient to compensate for the detrimental effects of visual disruption in people with CAI (Han et al., 2022). In addition, a previous meta-analysis, which aggregated data on time to boundary under both eyes-open and eyes-closed conditions among healthy controls and people with CAI, corroborated our findings by emphasizing the greater visual dependence among those with CAI (Song et al., 2016).

We posit that the impact of visual disruption on postural control in people with CAI stems from proprioceptive deficits. In situations of sensory deficits, proprioception can typically compensate for the loss of other sensory cues to maintain stability (Liu et al., 2023). However, people with CAI exhibit weaker proprioception, rendering them unable to provide sufficient and effective sensory information (Kawabata et al., 2024). This, in turn, leads to an inability to adequately compensate for the reduced visual inputs, ultimately resulting in compromised postural control (Redfern et al., 2001b). Conversely, people without CAI, who possess unimpaired proprioception, may exhibit reduced reliance on visual inputs and heightened reliance on proprioception in response to visual distractions.

The first limitation of this study is that, although stroboscopic glasses have been proven effective for visual disruption, they cannot simulate the complex visual changes associated with aging, such as macular degeneration, cataracts, and glaucoma, which affect visual perception. Therefore, future research should explore changes in physiological complexity, balance control, and injury potential in older adults with clinical vision loss. The second limitation lies in the gender imbalance in our participants, with a higher proportion of males, which may restrict the generalization of our findings to female populations. Future studies should strive to recruit a more gender-balanced participants to better represent the broader population and mitigate potential gender-related biases.

## 5 Conclusion

People with CAI are more vulnerable to visual disruption during static and dynamic postural control than those without CAI, suggesting that visual disruptions can adversely affect postural control in people with CAI, highlighting the importance of visual input in maintaining stable posture in this population.

## Data Availability

The datasets for this study can be found in the 4TU. ResearchData doi: 10.4121/9be48906-8440-44a2-8c7a-af630e6735de.v1.
